# Gut Microbiota Characteristics Are Associated With Severity of Acute Radiation-Induced Esophagitis

**DOI:** 10.3389/fmicb.2022.883650

**Published:** 2022-06-09

**Authors:** Ming-qiang Lin, Ya-hua Wu, Jun Yang, Han-cui Lin, Ling-yun Liu, Yi-lin Yu, Qi-wei Yao, Jian-cheng Li

**Affiliations:** ^1^College of Clinical Medicine for Oncology, Fujian Medical University, Fuzhou, China; ^2^Fujian Medical University Cancer Hospital, Fujian Cancer Hospital, Fuzhou, China

**Keywords:** acute radiation-induced esophagitis, gut microbiota, esophageal cancer, radiotherapy, 16S rRNA gene

## Abstract

**Background:**

Acute radiation-induced esophagitis (ARIE) is one of the most debilitating complications in patients who receive thoracic radiotherapy, especially those with esophageal cancer (EC). There is little known about the impact of the characteristics of gut microbiota on the initiation and severity of ARIE.

**Materials and Methods:**

Gut microbiota samples of EC patients undergoing radiotherapy (*n* = 7) or concurrent chemoradiotherapy (*n* = 42) were collected at the start, middle, and end of the radiotherapy regimen. Assessment of patient-reported ARIE was also performed. Based on 16S rRNA gene sequencing, changes of the gut microbial community during the treatment regimen and correlations of the gut microbiota characteristics with the severity of ARIE were investigated.

**Results:**

There were significant associations of several properties of the gut microbiota with the severity of ARIE. The relative abundance of several genera in the phylum Proteobacteria increased significantly as mucositis severity increased. The predominant genera had characteristic changes during the treatment regimen, such as an increase of opportunistic pathogenic bacteria including *Streptococcus*. Patients with severe ARIE had significantly lower alpha diversity and a higher abundance of *Fusobacterium* before radiotherapy, but patients with mild ARIE were enriched in *Klebsiella*, *Roseburia*, *Veillonella*, *Prevotella_9*, *Megasphaera*, and *Ruminococcus_2*. A model combining these genera had the best performance in prediction of severe ARIE (area under the curve: 0.907).

**Conclusion:**

The characteristics of gut microbiota before radiotherapy were associated with subsequent ARIE severity. Microbiota-based strategies have potential use for the early prediction of subsequent ARIE and for the selection of interventions that may prevent severe ARIE.

## Introduction

Acute radiation-induced esophagitis (ARIE) is one of the most common and debilitating tissue toxicities associated with radiotherapy in patients who received thoracic radiation, especially for esophageal cancer (EC) (Bradley and Movsas, 2004). ARIE is characterized by inflammation of the esophageal mucosa, with dysphagia, odynophagia, retrosternal pain, and other symptoms. The development of ARIE severely affects the quality-of-life of these patients. It also increases the need for narcotic analgesics and parenteral nutrition, and may even lead to treatment interruption or early treatment termination (Murro and Jakate, 2015). However, there are currently no effective non-invasive biomarkers or mucosal protective strategies for the management of ARIE.

The gut microbiota is the largest bank of microorganisms in the human body, the collective genes of which are 150-times larger than the human genome (Qin et al., 2010). Previous studies reported that gut microbiota can influence the occurrence and development of diseases in other organs *via* the gut-liver axis (Albillos et al., 2020), gut-brain axis (Cryan et al., 2019), gut-lung axis (Budden et al., 2017), and gut-bone axis (Zaiss et al., 2019). Recent research also demonstrated that patients with EC have altered gut microbiota (Deng et al., 2021).

Some studies suggested that the pathogenesis of radiotherapy- or chemotherapy-induced mucositis can be characterized by five major phases (Sonis, 2004) — initiation, upregulation, primary damage response, signal amplification, and ulceration — followed by wound healing. This five-phase model does not consider the role of gut microbiota in the pathophysiology of mucositis. However, with the development of high-throughput sequencing technology, increasing evidence has shown that commensal gut microbes play roles in several local and systemic inflammatory diseases, such as inflammatory bowel disease, radiation-induced enteritis, autoimmune disorders, allergic diseases, obesity, and diabetes (Clemente et al., 2018; Gerassy-Vainberg et al., 2018; Peroni et al., 2020; Scheithauer et al., 2020). Nevertheless, no study has yet reported the association of ARIE with the characteristics of gut microbiota.

Hence, we performed a longitudinal evaluation of the severity of ARIE in a cohort of patients with EC who received radiotherapy with or without chemotherapy, and examined fecal samples of these patients at the start, middle, and end of the radiotherapy regimen. Based on high-throughput 16s rRNA gene sequencing and bioinformatics analysis, we studied changes in the gut bacterial profile during the course of treatment, and examined the association between the severity of ARIE and the characteristics of the gut microbial community.

## Materials and Methods

### Subjects

This prospective cohort study was approved by the Ethics Committee of Fujian Cancer Hospital. Potential study subjects were identified from a group of newly diagnosed EC patients who received definitive radiotherapy with or without chemotherapy at our institution between August 2019 and July 2020. Informed consent was obtained from all included patients. The exclusion criteria were: chronic diarrhea, constipation, or a gastrointestinal disorder; prior receipt of chest radiation; and use of antibiotics or other drugs that could influence the gut microbiota within 1 month before study onset. A total of 49 EC patients were randomly recruited.

For all recruited patients, radiotherapy was administered 5 times per week at 200 cGy per fraction, with a total dose of 5000–6000 cGy over 5–6 weeks. Seven patients received radiotherapy alone, and 42 patients received paclitaxel (150 mg/m^2^) and nedaplatin (70 mg/m^2^) concurrent with radiotherapy. Chemotherapy was repeated every 21 days for two courses.

In all patients, esophageal mucositis was clinically evaluated once every 3 days by the same radiation therapist using the Radiation Therapy Oncology Group (RTOG) toxicity grading scale (Adebahr et al., 2016), which ranges from RTOG 0 (no symptoms) to RTOG 4 (severe symptoms). Patients with severe symptoms were provided analgesics, anti-inflammatory drugs, and parenteral nutrition support (if needed) in the later stage of treatment to relieve pain, prevent further deterioration of symptoms, and ensure the smooth completion of treatment. Fecal samples of all patients were collected at three time points: one day before the start of radiotherapy, at the middle of radiotherapy (2500–3000 cGy), and 1 day after the end of radiotherapy. In total, 147 fecal samples were collected for analysis.

### Sample Collection and 16s rRNA Gene Sequencing

Fresh feces were collected from each patient in a clean environment, added to an aseptic sampling tube, sent to the laboratory immediately, and then stored at -80°C. Total bacterial DNA was extracted using the Power Soil DNA Isolation Kit (MO BIO Laboratories) according to the manufacturer’s protocol. DNA quality and quantity were assessed by the ratios of A_260nm_/A_280nm_ and A_260nm_/A_230nm_. The V3–V4 region of the bacterial 16S rRNA gene was amplified using the common primer pair (forward, 5′- ACTCCTACGGGAGGCAGCA-3′; reverse, 5′- GGACTACHVGGGTWTCTAAT-3′), combined with adapter sequences and barcode sequences. Finally, all PCR products were quantified using the Quant-iT™ dsDNA HS Reagent and pooled together. High-throughput sequencing of bacterial rRNA genes was performed on the purified and pooled samples using the Illumina Hiseq 2500 platform (2 × 250 paired ends).

### Bioinformatics and Statistical Analysis

Paired and clean reads were merged as raw tags using FLASH (version 1.2.11) (Magoè and Salzberg, 2011). High-quality clean tags were obtained by Trimmomatic (version 0.33) (Bolger et al., 2014), and chimera checking was then performed using UCHIME (version 8.1) (Edgar et al., 2011). Operational Taxonomic Units (OTUs) that were clustered by effective tags were determined using USEARCH (version 10.0) (Edgar, 2013). For each representative sequence, the SILVA database (version 132) (Quast et al., 2013), based on the RDP classifier (version 2.2) algorithm (Wang et al., 2007), was used for annotation. Alpha diversity, measured from the Shannon and Chao1 indexes, was used to evaluate microbial richness, evenness, and community diversity. Mothur (version 1.30) was used to calculate these alpha diversity indexes and to generate rarefaction curve, rank abundance curve, and species accumulation boxplot (Schloss et al., 2009). Differences in alpha diversity were analyzed using the Wilcoxon rank-sum test. Beta-diversity, calculated using QIIME (version 1.9.1), was used to evaluate microbial community structure in different specimens. Based on the Beta-diversity of unweighted UniFrac metrics, data were visualized using principal coordinates analysis (PCoA). The significance of a difference in the microbial structure of groups was evaluated using a one-way analysis of similarity (ANOSIM). Statistical analyses of the diversity indexes and the relative abundance of genera were performed using R (version 4.1.1). Linear discriminant analysis (LDA) effect size (LEfSe) (Schloss et al., 2009) an algorithm for high-dimensional biomarker discovery, was used to determine the features most likely to explain differences between classes. An LDA threshold of 3.5 was used for discrimination. Spearman’s rank test was used for the correlation analyses, and data were visualized using the heatmap package of R.

Statistical analyses of the patients’ demographic characteristics and treatment-related factors were performed using SPSS (version 25.0). A one-way ANOVA was used for comparisons of continuous variables, and the Pearson Chi-square test or the Fisher exact test was for comparisons of categorical variables. All reported *p*-values are two-sided, and a *p*-value below 0.05 was considered significant.

## Results

Analysis of the baseline demographic characteristics of all 49 patients indicated that the groups with different ARIE severity were similar in terms of age, sex, BMI, tobacco use, alcohol use, comorbidities, pathology, radiation dose, clinical classification, or receipt of combined chemotherapy (Supplementary Table 1). The patients were 48–87 years old (average: 64.8), 79.59% were male, and 91.84% had squamous cell carcinoma. According to the eighth edition of the American Joint Committee on Cancer staging system for EC, the most common T classification was T3/T4 (77.55%) and the most common N classification was N0/N1 (69.39%). The median radiation dose was 6000 cGy and 42 patients (85.71%) received concurrent chemoradiotherapy. Analysis of the intervention treatments (use of analgesics, anti-inflammatory agents, and parenteral nutrition) showed significant differences among the groups.

In total, we generated 10,036,517 paired-end reads of high-quality sequences (average 68,276 per sample). There were 549 OTUs at a 97% similarity level. Analyses of the rarefaction and rank abundance curves showed that these curves tended flatten as the number of sample sequences increased, indicating the sample had sufficient richness and uniformity, and the sequencing depth was satisfactory (Supplementary Figures 1A,B). The species accumulation boxplot showed that the gene richness approached saturation as the sample size increased, indicating the number of samples was sufficient to resolve most of the genera present (Supplementary Figure 1C). These results thus indicated the sample size and sequencing depth were sufficient.

### Association of Acute Radiation-Induced Esophagitis Severity With Characteristics of the Microbial Community

We evaluated patients’ difficulties of swallowing or painful swallowing, eating, and body weight every 3 days according to the RTOG criteria throughout the entire radiotherapy regimen. And a series of fresh fecal samples from 49 patients were collected at the start, middle and end of the radiotherapy regimen, in which: 14 patients had no symptoms of ARIE (RTOG 0), 15 patients developed Grade 1 ARIE (RTOG 1), 13 patients developed Grade 2 ARIE (RTOG 2), and 7 patients developed Grade 3 ARIE (RTOG 3), furthermore, none of patients suffered Grade 4 ARIE (RTOG 4). We also observed that most patients with mucositis symptoms usually developed these symptoms by the end of the first week (1000 cGy), and an aggravation of symptoms during the middle of the radiotherapy regimen (2500–3000 cGy).

**FIGURE 1:**
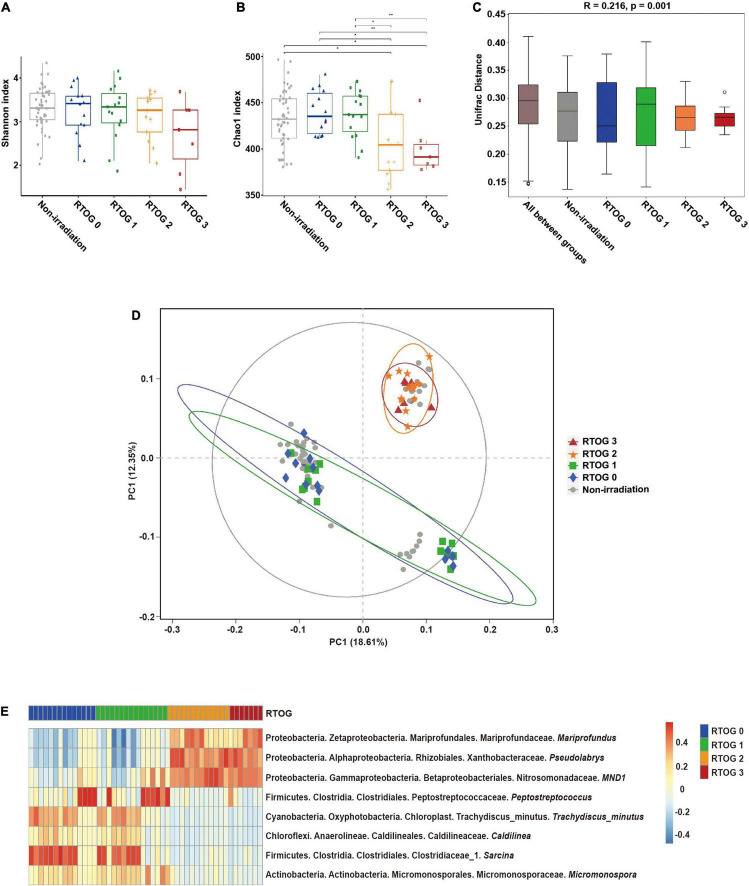
Relationship of ARIE severity with fecal microbial community structure of samples collected at “middle” of the radiotherapy regimen. **(A,B)** Alpha diversity indexes (Shannon and Chao1) of the non-irradiation (gray), RTOG 0 (blue), RTOG 1 (green), RTOG 2 (orange), and RTOG 3 (red) groups. A larger index indicates greater community diversity. **(C)** Box plot of inter-group and intra-group Unifrac distance-based ANOSIM. *R*-value > 0 indicated that the inter-group difference was greater than the intra-group difference. **(D)** PCoA of microbial community structure in the non-irradiation group and each RTOG group. Each sample is represented by a symbol, and symbols with different colors and shapes correspond to different groups. **(E)** Spearman correlation heatmap of the relationship of ARIE severity (RTOG grade) with the relative abundance of eight bacterial genera (| r| ≥ 0.5, *p* < 0.05), in which the relative abundance of each genus was converted to a log_10_ value, indicated by a color gradient. *P < 0.05; **P < 0.01.

To investigate the relationship of the fecal microbiota profile with the severity of mucositis, we classified all samples collected at the “start” of the radiotherapy regimen as a “non-irradiation group,” and classified samples collected at the “middle” of the radiotherapy regimen by RTOG grade (0, 1, 2, or 3), and then measured the Shannon and Chao1 indexes in these different samples. The Shannon index (an indicator of community richness and evenness) decreased as the RTOG grade increased from 0 to 3, but there were no statistically significant differences among these groups (Figure 1A). The Chao1 index (an indicator of community richness) was significantly different between the RTOG 1 and RTOG 2 groups (*p* = 0.023), but pair-wise comparisons indicated no significant differences in the non-irradiation, RTOG 0, and RTOG 1 groups, nor between the RTOG 2 and RTOG 3 groups (Figure 1B). A boxplot of ANOSIM analysis based on unweighted UniFrac distance indicated there were significant differences in the microbial communities of the different groups (*p* = 0.001, Figure 1C).

To visualize the microbial dissimilarity among these groups, we calculated the unweighted Unifrac distances for PCoA (Figure 1D). The results showed a clustering of the RTOG 0 and RTOG 1 groups, and of the RTOG 2 and RTOG 3 groups, indicating that the severity of ARIE was associated with the gut microbial community structure. We then tried to determine which types of bacteria were related to more severe ARIE (Figure 1E and Supplementary Figure 2). Spearman’s rank correlation analyses showed that the relative abundances of five genera had significantly negative correlations with RTOG grade — *Peptostreptococcus*, *Trachydiscus_minutus, Caldilinea*, *Sarcina, and Micromonospora*; the relative abundances of three genera had significantly positive correlations with RTOG grade — *Mariprofundus, Pseudolabrys*, and *MND1*.

Analysis of alpha diversity indexes between non-irradiation group and samples from four RTOG groups collected at the “end” of the radiotherapy regimen showed that the Shannon index was significantly different only between the non-irradiation group and RTOG 2 group (*p* = 0.0045). However, the Chao1 index of the non-irradiation group (*p* < 0.001), RTOG 0 group (*p* = 0.0028), and RTOG 1 group (*p* = 0.011) were all significantly different from that of the RTOG 2 group (Supplementary Figures 3A,B). A boxplot of ANOSIM analysis based on unweighted UniFrac distance also indicated significant differences in the microbial communities of the different groups (*p* = 0.001, Supplementary Figure 3C). A PCoA plot further indicated that the samples were clustered by groups (Supplementary Figure 3D). Notably, the Shannon and Chao1 indexes were both higher in the RTOG 3 group than the RTOG 2 group at this time. Although these differences were not statistically significant, it is possible that the receipt of anti-inflammatory and analgesic interventions after the “middle” of the radiotherapy regimen affected the structure of the gut microbiota.

We then investigated whether chemotherapy affected the microbial community during radiotherapy by comparing samples collected at the “middle” of the radiotherapy regimen from the 7 patients who received radiotherapy alone with the 42 patients who received chemoradiotherapy. The alpha diversity indexes (Shannon and Chao1) of these two groups were not significantly different (Supplementary Figure 4). Sixteen of the 42 patients (38.10%) who received chemoradiotherapy developed severe ARIE (RTOG 2-3), but 4 of 6 patients (66.67%) who received radiotherapy alone developed severe ARIE. However, Fisher’s exact test indicated no significant difference of these two groups, possibly due to the small sample size of the group that received radiotherapy alone.

### Changes in the Microbial Community During Chemoradiotherapy

**FIGURE 2:**
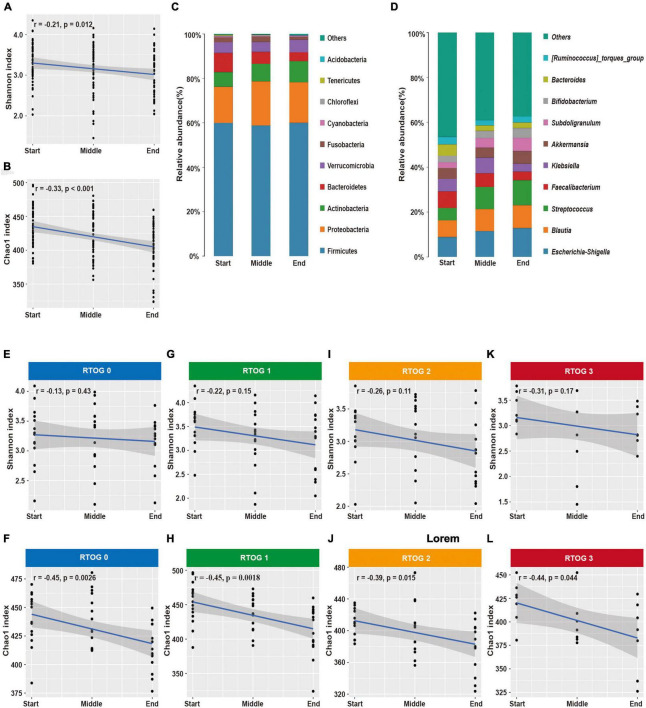
Changes in fecal microbial community structure during chemoradiotherapy. **(A,B)** Changes in alpha-diversity indexes (Shannon and Chao1) in the overall microbial community. **(C,D)** Average relative abundances of predominant bacterial taxa at the phylum and genus levels. Changes of alpha diversity indexes (Shannon and Chao1) in the RTOG 0 **(E,F)**, RTOG 1 **(G,H)**, RTOG 2 **(I,J)**, and RTOG 3 **(K,L)** groups during the course of chemoradiotherapy.

Our analysis of samples at the start, middle, and end of the radiotherapy regimen indicated there were significant decreases in the Shannon (*p* = 0.012) and Chao1 (*p* < 0.001) indexes over time (Figures 2A,B). In addition, we identified 10 major phyla in virtually all fecal samples — Firmicutes, Proteobacteria, Actinobacteria, Bacteroidetes, Verrucomicrobia, Fusobacteria, Cyanobacteria, Chloroflexi, Tenericutes, and Acidobacteria (Figure 2C and Supplementary Table 2). Throughout the treament regimen, the overall relative abundances of Actinobacteria and Acidobacteria increased, but the levels of Bacteroidetes and Tenericutes decreased. Ten genera accounted for nearly 60% of the total sequences at each sample time — *Escherichia-Shigella*, *Blautia*, *Streptococcus*, *Faecalibacterium*, *Klebsiella*, *Akkermansia*, *Subdoligranulum*, *Bifidobacterium*, *Bacteroides*, and *[Ruminococcus]_torques_group* (Figure 2D and Supplementary Table 2). Among them, the relative abundances of *Escherichia-Shigella*, *Blautia*, *Streptococcus*, *Subdoligranulum*, and *Bifidobacterium* increased over time, and the relative abundances of *Faecalibacterium* and *Bacteroides* decreased over time.

Because there were obvious changes in the fecal microbial community during chemoradiotherapy, we examined whether these changes were related to the severity of ARIE (Figures 2E–L). The Shannon and Chao1 indexes of each RTOG group declined as the treatment regimen progressed, but only the decreases of the Chao1 index were statistically significant (*p* = 0.0026, 0.0018, 0.015, 0.044, respectively). We also assessed the predominant bacterial genera in each individual patient over time (Supplementary Figure 5). Interestingly, changes in the relative abundance of each genus during the treatment regimen varied among patients, and these changes were unpredictable at an individual level. However, most patients had similar overall trends of increasing or decreasing relative abundances of specific genera.

### Fecal Microbiota Biomarkers for Radiation-Induced Esophagitis Severity

Given that changes in the relative abundances of the predominant fecal bacterial genera had similar trends in most patients during the course of treatment, we then sought to identify whether the structure of the microbial community before treatment was related to subsequent ARIE severity. A total of 29 patients (59.18%) only experienced mild ARIE (RTOG 0–1) and 20 patients (40.82%) developed severe ARIE (RTOG 2–3) after receiving roughly the same cumulative radiation dose. Our analysis of samples collected at the “middle” of the radiotherapy regimen indicated the Chao1 index was significantly decreased in the RTOG 1 group relative to the RTOG 2 group (*p* = 0.023); however, there were no significant differences between the RTOG 0 and RTOG 1 groups, nor between the RTOG 2 and RTOG 3 groups (Figure 1B). Besides, the PCoA plot showed a clustering of the RTOG 0 and 1 groups, and of the RTOG 2 and 3 groups (Figure 1D). Similarly, the PCoA plot of samples collected at the “start” of the radiotherapy regimen further confirmed the pairwise clustering of the RTOG groups (Supplementary Figure 6). We therefore divided all 49 patients into a mild ARIE subgroup (RTOG 0–1) and a severe ARIE subgroup (RTOG 2–3). Supplementary Table 3 summarizes the demographic characteristics and treatments-related factors of these two groups.

**FIGURE 3:**
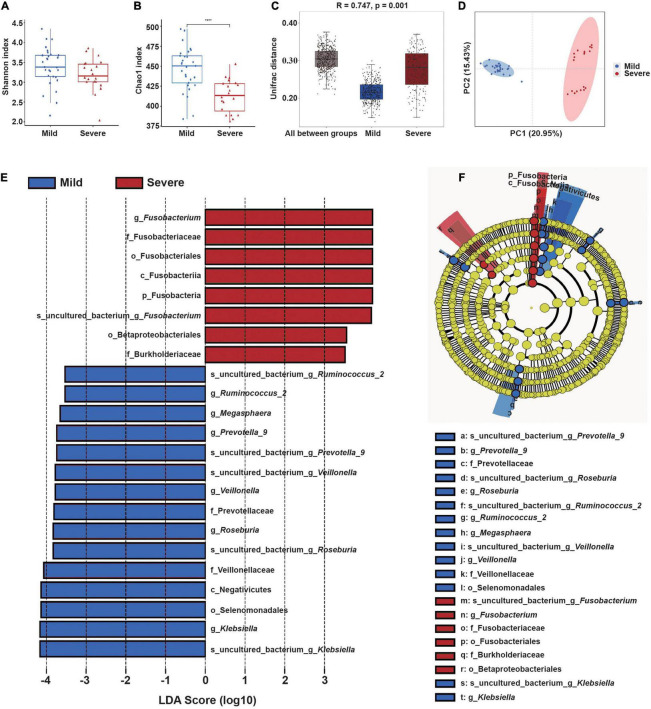
Gut microbial community structure before radiotherapy (“start”) in patients who subsequently experienced mild or severe ARIE. **(A,B)** Alpha-diversity indexes (Shannon and Chao1) of the mild (blue) and severe (red) subgroups. **(C)** Box plot of inter-group and intra-group Unifrac distance-based ANOSIM. *R*-value > 0 indicated the inter-group difference was greater than the intra-group difference. **(D)** PCoA of Unifrac distance of the mild and severe subgroups. **(E,F)** LEfSe analysis (LDA significance threshold > 3.5) of taxa with the greatest differences in abundance between the severe (red, positive scores) and mild (blue, negative scores) subgroups. ****P < 0.0001.

We then compared the microbial community structure of these two subgroups before radiotherapy, using samples collected at the “start” of the radiotherapy regimen (Figures 3A,B). The results showed the Chao1 index of the severe ARIE group was significantly lower than that of the mild ARIE group (*p* < 0.001), but these two groups had no significant difference in the Shannon index. Thus, the mild ARIE group harbored a greater community richness of fecal microbiota than the severe ARIE group. PCoA revealed a clear separation of microbial community structure of these two groups, and ANOSIM analysis based on unweighted UniFrac distance also showed significant differences between these two groups (Figures 3C,D). To further determine the specific bacterial taxa which were distinct between these two groups, we compared the composition of fecal microbiota using LEfSe (Figures 3E,F). The results indicated that 23 taxa had different abundances in the mild and severe groups, including 7 taxa at the genus level. Based on LDA effect size, *Fusobacterium* was enriched in the severe ARIE group, *Klebsiella*, *Roseburia*, *Veillonella*, *Prevotella_9*, *Megasphaera*, and *Ruminococcus_2* were enriched in the mild ARIE group. Analysis of the changes in the relative abundances of these seven taxa during chemoradiotherapy indicated there were downward trends in the mild and severe ARIE groups (Supplementary Figure 7).

**FIGURE 4:**
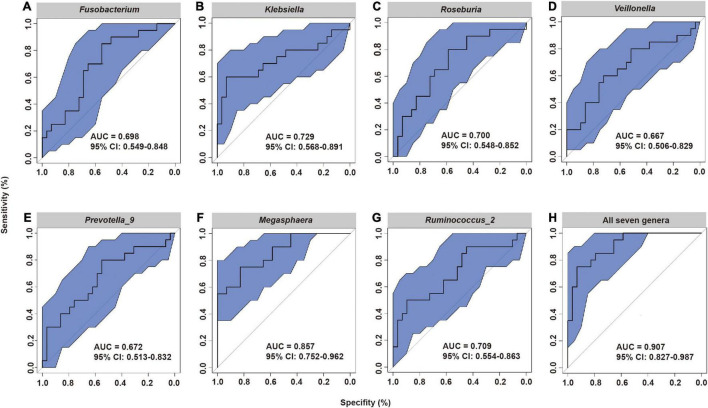
Receiver operating characteristic (ROC) analysis of the predictive value of the abundance of seven fecal microbial genera prior to radiotherapy on subsequent severity of ARIE. **(A)** *Fusobacterium*, **(B)** *Klebsiella*, **(C)** *Roseburia*, **(D)** *Veillonella*, **(E)** *Prevotella_9*, **(F)** *Megasphaera*, **(G)** *Ruminococcus_2*, **(H)** All seven genera.

These results led us to hypothesize that the specific fecal bacterial taxa present before radiotherapy may affect the severity of subsequent ARIE. To test this hypothesis, we used receiver-operating-characteristic (ROC) analysis to evaluate the predictive value of bacteria that were statistically significant in the LEfSe analysis (Figures 4A–G). The results showed that the area under the curve (AUC) ranged from 0.667 [95% confidence interval (CI): 0.506–0.829] for *Veillonella* to 0.857 (95% CI: 0.752–0.962) for *Megasphaera*. Moreover, the combined use of all seven genera increased the AUC to 0.907 (95% CI: 0.827–0.987, Figure 4H), indicating that the composition of the fecal microbial community before radiotherapy can provide excellent prediction of the subsequent severity of ARIE.

### Association of Fecal Bacteria With Indicators of Clinical Immune-Inflammation

**FIGURE 5:**
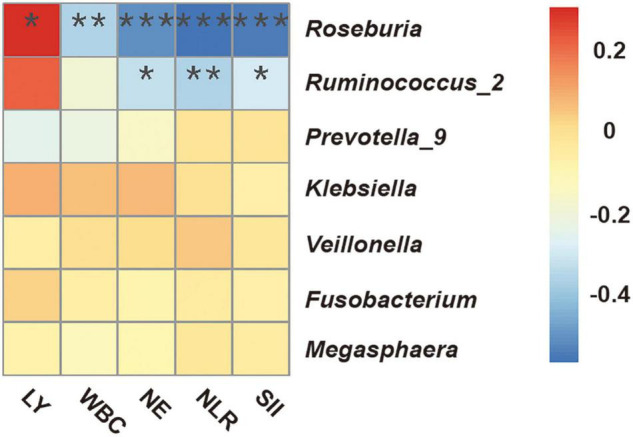
Spearman correlation heatmap between fecal microbial genera and clinical indicators of immune-inflammation before radiotherapy (“start”). WBC, white blood cells; NE, neutrophils; LY, lymphocytes; NLR, neutrophil-lymphocyte ratio; SII, systemic immune inflammation index (platelets × NLR). *P < 0.05; **P < 0.01; ***P < 0.001.

We performed Spearman correlation analysis to evaluate the potential relationship of the candidate bacterial genera with multiple indicators of clinical immune-inflammation collected at the “start” of the radiotherapy regimen (Figure 5): lymphocytes (LY), white blood cells (WBC), neutrophils (NE), neutrophil-lymphocyte ratio (NLR), and systemic immune inflammation index (SII; platelets × NLR). The results indicated significant correlations for two short-chain fatty acid-producing bacteria — *Roseburia* (all 5 indicators) and *Ruminococcus_2* (3 indicators). *Roseburia* was positively correlated with LY, and negatively correlated with WBC, NE, NLR, and SII; *Ruminococcus_2* was also negatively correlated with NE, NLR and SII. These results indicated that the presence of specific fecal bacteria before radiotherapy was related to the systemic immune inflammatory state at that time.

## Discussion

Esophageal cancer is the sixth leading cause of cancer-related deaths and has the seventh highest of incidence of all cancers worldwide (Sung et al., 2021). Most patients have locally advanced disease at diagnosis, and radiotherapy is one of the most important therapeutic modalities for these patients (Ajani et al., 2019). The esophagus consists of rapidly proliferating squamous epithelial cells that course longitudinally through the entire extent of the mediastinum, and is highly vulnerable to radiation-induced injury. ARIE remains the main dose-limiting factor for radiotherapy of patients with EC. Most studies of ARIE have focused on radiotherapy dose-volume parameters (Caglar et al., 2010; Werner-Wasik et al., 2010). However, no previous research investigated the possible role of gut microbiota on the development of ARIE.

In addition to the widely studied effect of the gut microbiota on radiation-induced enteritis (Gerassy-Vainberg et al., 2018), recent studies confirmed that gut microbiota also regulates radiation-induced injury of extraintestinal organs, such as cardiopulmonary and hematopoietic damage (Chen et al., 2021; Kordahi and Chassaing, 2021). Analysis of samples collected at the “middle” of the radiotherapy regimen, that is, when ARIE occurred, showed that the RTOG 0 and RTOG 1 groups both had greater gut microbial richness than the RTOG 2 and RTOG 3 groups, and a PCoA plot also showed that the samples were clustered by groups. This indicated that the severity of ARIE was associated with the gut microbial community structure. Further analysis indicated that all genera which increased significantly as mucositis severity increased were in the phylum Proteobacteria, and that several members of the phyla Firmicutes, Cyanobacteria, Chloroflexi, and Actinobacteria decreased significantly as mucositis severity increased. A systematic literature review reported that an imbalanced gut microbiota often arises from a sustained increase in the abundance of bacteria in the phylum Proteobacteria, and the natural human gut microbiota normally contains only a minor proportion bacteria of this phylum (Shin et al., 2015). Although most of the bacteria that had high correlations with mucositis severity had relatively low abundances, they could still play major roles in causing ARIE. In particular, the “keystone pathogen” hypothesis (Hajishengallis et al., 2012) proposes that certain low-abundance bacteria, such as *Porphyromonas gingivalis* in the human periodontium or enterotoxigenic *Bacteroides fragilis* in the human colon, can provoke inflammation by remodeling the entire microbiota *via* direct effects on different microbes or indirect effects due to host modulation.

Previous research showed that pelvic radiation induced gut dysbiosis, and that the altered gut microbiota increased the susceptibility to inflammation (Gerassy-Vainberg et al., 2018). This raises the question of whether chest radiation can also alter gut microbiota. We therefore collected patients’ feces longitudinally during the radiotherapy regimen to examine the changes in their gut microbiota. Our results showed that Chao1 indexes decreased significantly as the treatment progressed, indicating a decline in community richness of gut microbiota. It is worth noting that the majority of patients in our study (42/49) received chemotherapy concurrent with radiotherapy. Previous studies found that chemotherapy-induced gut microbiota dysbiosis can impair mucosal homeostasis (van Vliet et al., 2010; Wei et al., 2021). However, we found no significant difference in the alpha diversity indexes (Shannon and Chao1) of gut microbiota in the mid-treatment samples of patients who received chemoradiotherapy relative to those who received radiotherapy alone. Fisher’s exact test also confirmed that the incidence of severe ARIE was not statistically different between these two groups, although only 7 patients were in the group that received radiotherapy alone. It is generally believed that a stable and diverse gut microbiota is essential to the normal physiological processes and mucosal immune function of the host. Normal microbiota can prevent colonization by foreign pathogens and the overgrowth of local pathogens, and an altered composition of commensal microbes may cause immune imbalance and increase the risk of multiple diseases (Kayama et al., 2020).

Twenty of the 49 patients (40.82%) in our study developed severe ARIE, comparable to the incidence of severe ARIE in EC patients who received chemoradiotherapy in previous studies (Crosby et al., 2013; Yu et al., 2021). We hypothesized that the distribution of gut microbiota prior to radiotherapy determines a patient’s susceptibility to mucosal radiation injury. Our analysis of fecal samples of all patients prior to radiotherapy indicated that the group which subsequently developed the severe ARIE had a lower initial richness of gut microbiota. Furthermore, compared to the mild ARIE subgroup, fecal samples from the severe ARIE subgroup had significantly higher abundances of *Fusobacterium* and Burkholderiaceae, both of which are associated with harmful infections in humans. In particular, *Fusobacterium* has the ability to produce a variety of virulence factors and *Fusobacterium nucleatum*, one of the most prevalent species isolated from human infections, has been implicated in atherosclerosis, adverse pregnancy outcomes, rheumatoid arthritis, and organ abscesses and infections (Han, 2015; Brennan and Garrett, 2019). Mechanistic studies of *F. nucleatum* showed it promoted nuclear factor kappa-light-chain-enhancer of activated B cell (NF-κB) activation, and indirectly led to a proinflammatory genetic signature that included increased expression of interleukin-1β (IL-1β), interleukin-6 (IL-6), interleukin-8 (IL-8), and tumor necrosis factor-α (TNFα) (Kostic et al., 2013). A previous study demonstrated that *F. nucleatum* secreted outer membrane vesicles (OMVs) (Liu et al., 2019), “buds” of the outer membrane that typically contain antigenic components that can activate Toll-like receptors (TLRs) on epithelial cells or immune cells. TLR activation is also linked to the activation of downstream targets, such as extracellular signal-regulated kinase (ERK), cAMP-response element binding protein (CREB), and NF-κB. Fusobacteria also encode Fusobacterium adhesin A (FadA), which likely provides it with the ability to invade epithelial and endothelial cells, and increase the permeability of the endothelial layer (Han, 2015). The Burkholderiaceae is a well-known family of heterotrophic bacteria that can colonize the guts of immunosuppressed patients, and this family includes species associated with several animal and human diseases of varying severity (Yang et al., 2016; Ye et al., 2021). Further research is needed to identify the exact pathogenic species of *Fusobacterium* and Burkholderiaceae that are responsible for the effects observed here.

We found that patients in the mild ARIE subgroup had significantly higher abundances of *Roseburia*, *Veillonella*, *Prevotella_9*, *Megasphaera*, and *Ruminococcus_2*, genera associated with the production of short-chain fatty acids (SCFAs) (de la Cuesta-Zuluaga et al., 2017; Louis and Flint, 2017; D’Amico et al., 2021), including butyrate, propionate, and acetate. Many studies have examined butyrate due to its anti-inflammatory and anti-cancer effects. In particular, butyrate is a main energy source for normal epithelial cells, but not for cancer cells (Vatanen et al., 2016a). Interestingly, previous research reported that the presence of *Fusobacterium* spp. in the gut microbiota was associated with reduced butyrate production (Hertel et al., 2021). Researchers have proposed two main mechanisms for the anti-inflammatory and immunomodulatory effects of butyrate. First, butyrate can inhibit the phosphorylation of the NF-κB p65 signaling pathway in host immune cells by binding to G-protein-coupled receptors (GPR43, GPR41, and GPR109A), thereby blocking inflammatory responses and suppressing the release of TNF-α and IL6 (Lewis et al., 2010). Second, butyrate can inhibit histone deacetylase (HDAC), thus increasing Forkhead box protein P3 (FOXP3) expression and inducing Treg cell polarization; these cells are important producers of IL-10, a potent anti-inflammatory cytokine (Furusawa et al., 2013). Butyrate also assists in maintaining the gut barrier function by regulating the expression of mucin 2 and tight-junction proteins, such as claudin-1 and Zonula Occludens-1, which prevent bacterial and lipopolysaccharide (LPS) translocation and systemic inflammation (Tajik et al., 2020; Zhang et al., 2021). It is also important that SCFAs can mediate a range of extra-intestinal effects and impact development of immune and inflammatory responses, both locally and at distant sites, after entering the circulation *via* active transport mediated by monocarboxylate transporters (Stumpff, 2018). The therapeutic potential of butyrate and other SCFAs for the management of ARIE should therefore be considered in further research.

Notably, we found that *Klebsiella* was also enriched in the fecal samples of patients in the mild ARIE subgroup. The LPS of *Klebsiella* is generally considered to be a toxin that promotes inflammation *via* TLR4-mediated NF-κB activation and the production of various inflammatory factors, such as TNF-α, IL-6, and IL-1β. However, Vatanen et al. (2016b) suggested that the lipid A domain of LPS, which has six acyl chains (produced by Enterobacteriaceae, including *Klebsiella*), may be a beneficial innate immune activator. Initial exposure to LPS from Enterobacteriaceae prevented TNF-α production after restimulation, referred to as induction of endotoxin tolerance. In contrast, the lipid A domain of LPS, which has four or five acyl chains (produced by Bacteroides), may inhibit innate immune activation and endotoxin tolerance (Vatanen et al., 2016a; D’Hennezel et al., 2017). Based on microbial signatures, we constructed ROC curves to examine genera that have potential for predicting the severity of ARIE. Notably, models that examined a single genus or all seven genera provided reliable predictions of severe ARIE (RTOG 2–3).

Previous research showed that secretion of IL-6 and TNF-α amplified leukocyte activation, and led to increased levels of WBCs and NEs (Fujishima and Aikawa, 1995). The NLR and LY level are also good indicators of bacteremia (de Jager et al., 2010). Recent research proposed use of the SII (platelets × NLR) as an integrated and novel inflammatory biomarker (Mazza et al., 2020). Researchers initially used the SII to assess the prognosis of patients with solid cancers and coronary heart disease, and now also use it as a measure of inflammation status. We found that *Roseburia* and *Ruminococcus_2* had significant correlations with these indicators of clinical immune-inflammation. This supports the presence of potential relationships among the characteristics of gut microbiota, ARIE, and the systemic inflammatory response.

Our study identified an association between the characteristics of the gut microbial community of EC patients and the severity of ARIE, as well as changes of the gut microbial structure during chemoradiotherapy. We suggest that the pre-existing gut microbial community in these patients may be useful in predicting which patients are more likely to progress to severe ARIE. This study represents, as far as we know, the largest longitudinal cohort study of the association of human gut microbiota with ARIE, but future studies with larger sample sizes are needed to verify our results. Furthermore, we suggest a shotgun microbiota metagenomics approach to better understand the structure and function of the gut microbial community at the species level. Germ-free and gnotobiotic animals are also invaluable experimental tools for examination of host-microbe interactions.

## Conclusion

In summary, our results showed that the characteristics of the gut microbial community of EC patients prior to radiotherapy correlated with subsequent ARIE severity. Microbiota-based strategies may be useful for the early prediction and prevention of severe ARIE of patients with EC.

## Data Availability Statement

The datasets presented in this study can be found in online repositories. The names of the repository/repositories and accession number(s) can be found below: FigShare, https://figshare.com/articles/dataset/allsample_txt/19235931; doi:10.6084/m9.figshare.19235931.

## Ethics Statement

The current study was approved by the Ethics Committee of Fujian Medical University Cancer Hospital, Fuzhou, China (K2022-008-01). The patients/participants provided their written informed consent to participate in this study.

## Author Contributions

J-CL, Q-WY, and M-QL conceived the idea. M-QL, Y-HW, and H-CL performed the experiments. JY, L-YL, and Y-LY analyzed the data. M-QL wrote the manuscript. J-CL, Q-WY, and Y-HW revised the manuscript. All authors helped to perform the research.

## Conflict of Interest

The authors declare that the research was conducted in the absence of any commercial or financial relationships that could be construed as a potential conflict of interest.

## Publisher’s Note

All claims expressed in this article are solely those of the authors and do not necessarily represent those of their affiliated organizations, or those of the publisher, the editors and the reviewers. Any product that may be evaluated in this article, or claim that may be made by its manufacturer, is not guaranteed or endorsed by the publisher.

## Abbreviations

ARIE: acute radiation-induced esophagitis
AUC: area under the curve
ANOSIM: one-way analysis of similarity
CI: confidence interval
CREB: cAMP-response element binding protein
EC: esophageal cancer
ERK: extracellular signal-regulated kinase
FadA: Fusobacterium adhesin A
FOXP3: forkhead box protein P3
GPR: G-protein-coupled receptors
HDAC: histone deacetylase
IL-1β: interleukin-1β
IL-6: interleukin-6
IL-8: interleukin-8
LDA: linear discriminant analysis
LEfSe: linear discriminant analysis effect size
LPS: lipopolysaccharide
LY: lymphocytes
NE: neutrophils
NLR: neutrophil-lymphocyte ratio
NF-κB: nuclear factor kappa-light-chain-enhancer of activated B cell
OTUs: operational taxonomic units
OMVs: outer membrane vesicles
PCoA: principal coordinates analysis
RTOG: Radiation Therapy Oncology Group
ROC: receiver-operating-characteristic
SCFAs: short-chain fatty acids
SII: systemic immune inflammation index
TLRs: Toll-like receptors
TNFα: tumor necrosis factor-α
WBC: white blood cells.
