# Supplementary material for: Gut Microbiota Characteristics Are Associated With Severity of Acute Radiation-Induced Esophagitis
Source: Front Microbiol. 2022 Jun 9;13:883650. doi: 10.3389/fmicb.2022.883650 (PMC9218355; doi:10.3389/fmicb.2022.883650)
Supplement: Supplementary file 1 [file Data_Sheet_1.zip › Datasheet 1.docx]

**Supplementary Table 1.** Patient Characteristics, Treatments, and ARIE Grades

| **Variable** | **RTOG 0 (n=14)** | **RTOG 1 (n=15)** | **RTOG 2 (n=13)** | **RTOG 3 (n=7)** | **p** | |
| --- | --- | --- | --- | --- | --- | --- |
| Age (years) |  |  |  |  | 0.544 | |
| Mean ± SD | 65.71±9.09 | 62.87±9.00 | 67.46±11.88 | 62.14±8.65 |  | |
| Median | 67.00 | 63.50 | 68.00 | 62.00 |  | |
| Sex |  |  |  |  | 0.960 | |
| Male | 11 | 12 | 11 | 5 |  | |
| Female | 3 | 3 | 2 | 2 |  | |
| BMI |  |  |  |  | 0.563 | |
| Mean ± SD | 20.98±2.33 | 22.36±3.40 | 21.80±2.39 | 22.22±2.34 |  | |
| Median | 21.71 | 22.21 | 22.04 | 22.49 |  | |
| Tobacco use |  |  |  |  | 0.069 | |
| No | 13 | 8 | 7 | 5 |  | |
| Yes | 1 | 7 | 6 | 2 |  | |
| Alcohol use |  |  |  |  | 0.196 | |
| No | 13 | 9 | 10 | 6 |  | |
| Yes | 1 | 6 | 3 | 1 |  | |
| Comorbidities |  |  |  |  | 0.528 | |
| No | 7 | 10 | 10 | 4 |  | |
| Yes | 7 | 5 | 3 | 3 |  | |
| Pathology |  |  |  |  | 0.820 | |
| Sq. cell carcinoma | 12 | 14 | 12 | 7 |  | |
| Adenocarcinoma | 2 | 1 | 1 | 0 |  | |
| Radiation dose (cGy) |  |  |  |  | 0.974 | |
| Mean ± SD | 5758.21±442.58 | 5746.00±439.67 | 5686.92±423.62 | 5705.71±469.57 |  |  |
| Median | 6000.00 | 6000.00 | 5940.00 | 5940.00 |  | |
| T classification |  |  |  |  | 0.549 | |
| 1-2 | 4 | 4 | 3 | 0 |  | |
| 3-4 | 10 | 11 | 10 | 7 |  | |
| N classification |  |  |  |  | 1.000 | |
| 0-1 | 10 | 10 | 9 | 5 |  | |
| 2-3 | 4 | 5 | 4 | 2 |  | |
| M classification |  |  |  |  | 0.820 | |
| 0 | 12 | 14 | 12 | 7 |  | |
| 1 | 2 | 1 | 1 | 0 |  | |
| Chemotherapy |  |  |  |  | 0.673 | |
| No | 2 | 1 | 3 | 1 |  | |
| Yes | 12 | 14 | 10 | 6 |  | |
| Analgesics |  |  |  |  |  | |
| No | 14 | 12 | 6 | 2 | 0.001 | |
| Yes | 0 | 3 | 7 | 5 |  | |
| Anti-inflammatory |  |  |  |  | < 0.001 | |
| No | 14 | 15 | 11 | 0 |  | |
| Yes | 0 | 0 | 2 | 7 |  | |
| Parenteral nutrition |  |  |  |  |  | |
| No | 14 | 15 | 11 | 4 | 0.004 | |
| Yes | 0 | 0 | 2 | 3 |  | |

**Supplementary Table 2.** Average relative abundances of predominant bacterial taxa

| **Taxa** | | **Start** | **Middle** | **End** |
| --- | --- | --- | --- | --- |
| **Phylum** |  |  |  |  |
| Firmicutes | 0.5994665 | 0.58786375 | 0.59993674 |  |
| Proteobacteria | 0.16257513 | 0.1991137 | 0.18251873 |  |
| Actinobacteria | 0.06678453 | 0.07832828 | 0.09516772 |  |
| Bacteroidetes | 0.08601804 | 0.05436669 | 0.03979767 |  |
| Verrucomicrobia | 0.04791594 | 0.0442511 | 0.05665526 |  |
| Fusobacteria | 0.02218944 | 0.02474316 | 0.01285367 |  |
| Cyanobacteria | 0.00415515 | 0.00229459 | 0.00498082 |  |
| Chloroflexi | 0.00340166 | 0.00401215 | 0.00133565 |  |
| Tenericutes | 0.00388865 | 0.00108302 | 0.00085403 |  |
| Acidobacteria | 0.00064904 | 0.00135901 | 0.00238195 |  |
| **Genus** |  |  |  |  |
| *Escherichia-Shigella* | 0.08885447 | 0.11489596 | 0.12846158 |  |
| *Blautia* | 0.0745369 | 0.09790686 | 0.10224759 |  |
| *Streptococcus* | 0.05468358 | 0.09887817 | 0.11123499 |  |
| *Faecalibacterium* | 0.07406538 | 0.06096133 | 0.03791138 |  |
| *Klebsiella* | 0.05584282 | 0.07001156 | 0.03531516 |  |
| *Akkermansia* | 0.04791594 | 0.0442511 | 0.05665526 |  |
| *Subdoligranulum* | 0.02590745 | 0.04261363 | 0.05850174 |  |
| *Bifidobacterium* | 0.0288982 | 0.03167458 | 0.04391878 |  |
| *Bacteroides* | 0.05134768 | 0.02522779 | 0.02456509 |  |
| *[Ruminococcus]_torques_group* | 0.03260718 | 0.02324943 | 0.02609946 |  |

**Supplementary Table 3.** Patient Characteristics, Treatments, and ARIE Severity

| **Variable** | **Mild (n=29)** | **Severe (n=20)** | **p** |
| --- | --- | --- | --- |
| Age (years) |  |  | 0.636 |
| Mean ± SD | 64.24±9.00 | 65.60±10.93 |  |
| Median | 66.00 | 65.00 |  |
| Sex |  |  | 1.000 |
| Male | 23 | 16 |  |
| Female | 6 | 4 |  |
| BMI |  |  | 0.753 |
| Mean ± SD | 21.69±2.96 | 21.94±2.32 |  |
| Median | 22.06 | 22.26 |  |
| Tobacco use |  |  | 0.362 |
| No | 21 | 12 |  |
| Yes | 8 | 8 |  |
| Alcohol use |  |  | 1.000 |
| No | 22 | 16 |  |
| Yes | 7 | 4 |  |
| Comorbidities |  |  | 0.417 |
| No | 17 | 14 |  |
| Yes | 12 | 6 |  |
| Pathology |  |  | 0.636 |
| Sq. cell carcinoma | 26 | 19 |  |
| Adenocarcinoma | 3 | 1 |  |
| Radiation dose (cGy) |  |  | 0.643 |
| Mean ± SD | 5751.90±433.17 | 5693.50±427.85 |  |
| Median | 6000.00 | 5940.00 |  |
| T classification |  |  | 0.491 |
| 1-2 | 8 | 3 |  |
| 3-4 | 21 | 17 |  |
| N classification |  |  | 0.938 |
| 0-1 | 20 | 14 |  |
| 2-3 | 9 | 6 |  |
| M classification |  |  | 0.636 |
| 0 | 26 | 19 |  |
| 1 | 3 | 1 |  |
| Chemotherapy |  |  | 0.422 |
| No | 3 | 4 |  |
| Yes | 26 | 16 |  |
| Analgesics |  |  | < 0.001 |
| No | 26 | 8 |  |
| Yes | 3 | 12 |  |
| Anti-inflammatory |  |  | < 0.001 |
| No | 29 | 11 |  |
| Yes | 0 | 9 |  |
| Parenteral nutrition |  |  |  |
| No | 29 | 15 | 0.008 |
| Yes | 0 | 5 |  |
