# Supplementary material for: Gut Microbiota Characteristics Are Associated With Severity of Acute Radiation-Induced Esophagitis
Source: Front Microbiol. 2022 Jun 9;13:883650. doi: 10.3389/fmicb.2022.883650 (PMC9218355; doi:10.3389/fmicb.2022.883650)
Supplement: Supplementary file 1 [file Data_Sheet_1.zip › Supplementary Figure Legends.docx]

**Supplementary figure legends**

**Figure S1. Data quality of bacterial 16s rRNA gene sequences.** (A) Rarefaction curve. (B) Rank abundance curve. (C) Species accumulation boxplot.

**Figure S2. Association of f****ecal bacterial genera with the severity of ARIE (RTOG grade; |r| ≥ 0.5, P < 0.05).**

**Figure S3. Relationship of ARIE severity with fecal microbial community structure of samples collected at the “end” of the radiotherapy regimen.** (A, B) Alpha diversity indexes (Shannon and Chao1) of the non-irradiation (grey), RTOG 0 (blue), RTOG 1 (green), RTOG 2 (orange), and RTOG 3 (red) groups. A larger index indicates greater community diversity. (C) Box plot of inter-group and intra-group Unifrac distance-based ANOSIM. R-value > 0 indicated that the inter-group difference was greater than the intra-group difference. (D) PCoA of microbial community structure in the non-irradiation group and each RTOG group. Each sample is represented by a symbol, and symbols with different colors and shapes correspond to different groups. *P<0.05; **P<0.01; ****P<0.0001.

**Figure S4. Alpha-diversity of gut microbiota in patients receiving radiotherapy alone *vs.* chemoradiotherapy.** (A) Shannon index. (B) Chao1 index.

**Figure S5. Changes in the relative abundances of the predominant genera in 49 EC patients during the course of chemoradiotherapy.** Each colored line represents the relative abundance of a specific genus (*Escherichia-Shigella*, *Blautia*, *Streptococcus*, *Faecalibacterium*, *Klebsiella*, *Akkermansia*, *Subdoligranulum*, *Bifidobacterium*, *Bacteroides* or *[Ruminococcus]_torques_group*) in a single patient during the treatment regimen.

**Figure S6. PCoA of microbial community structure of samples collected at the “start” of the radiotherapy regimen.** Each sample is represented by a symbol, and symbols with different colors and shapes correspond to different groups.

**Figure S7. Changes in the relative abundance of differential taxa obtained by LEfSe analysis in the mild and severe ARIE groups during chemoradiotherapy.**
