# Supplementary figures and images for: Gut Microbiota Characteristics Are Associated With Severity of Acute Radiation-Induced Esophagitis
Source: Front Microbiol. 2022 Jun 9;13:883650. doi: 10.3389/fmicb.2022.883650 (PMC9218355; doi:10.3389/fmicb.2022.883650)

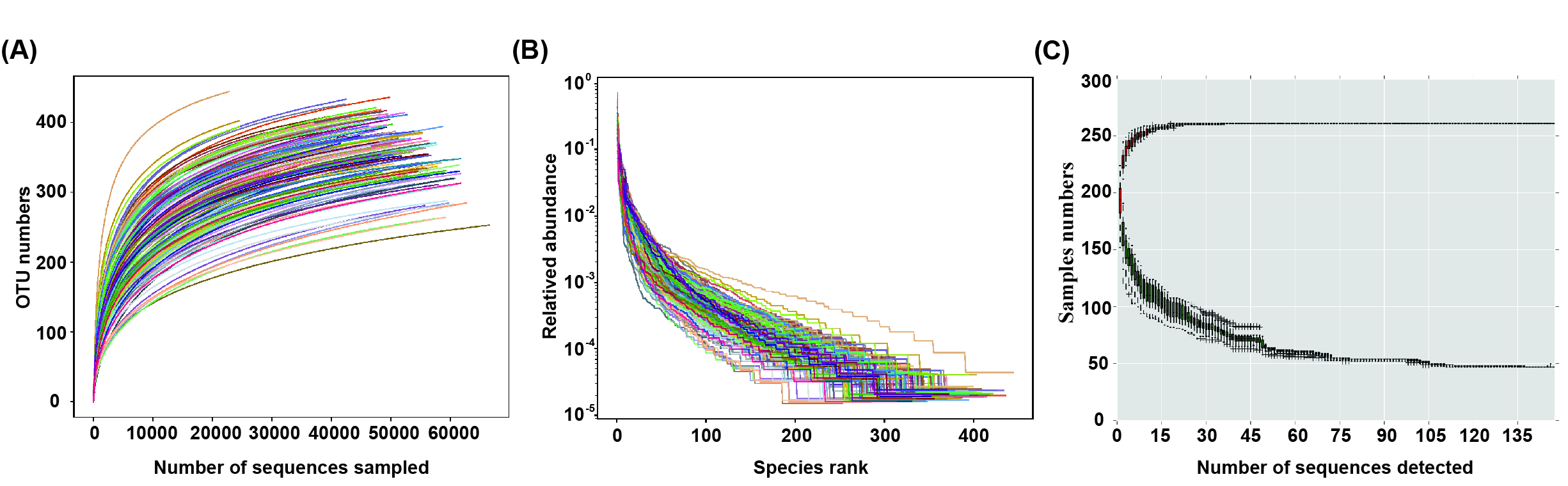

Supplement: Supplementary file 2 [file Image_1.JPEG]

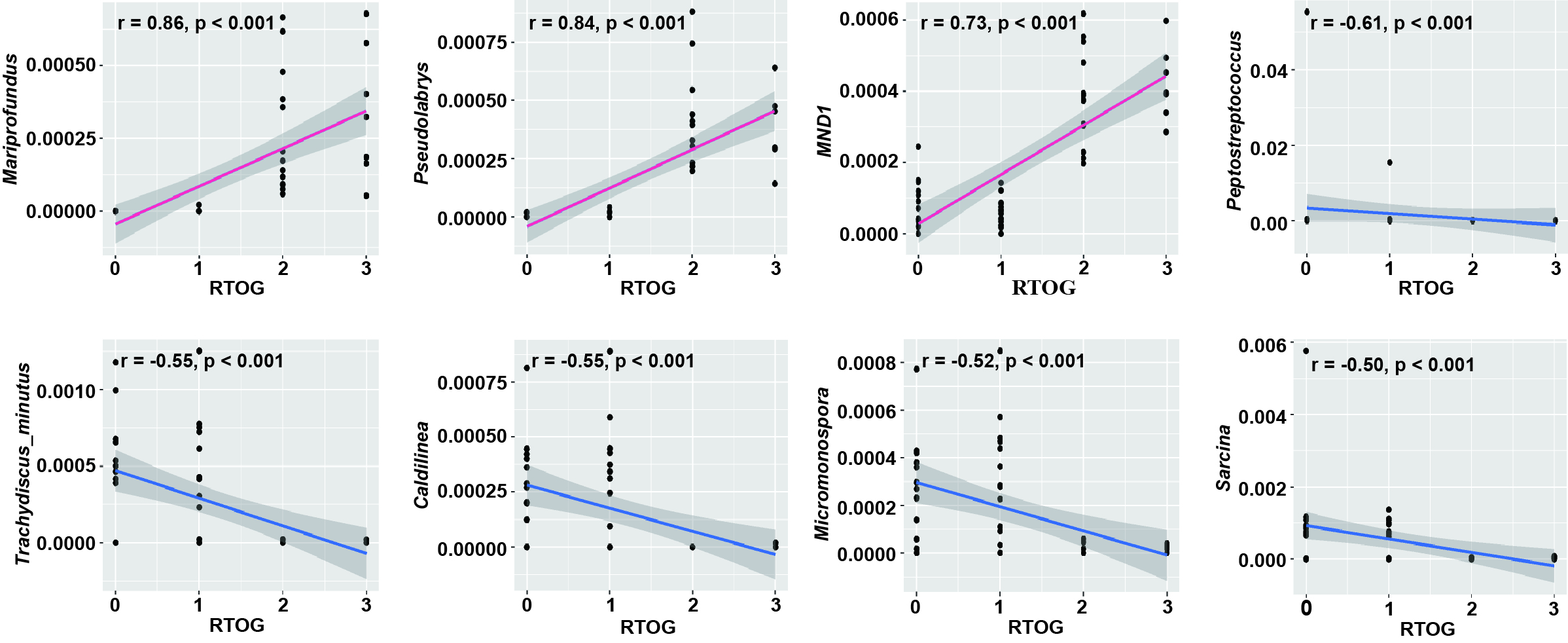

Supplement: Supplementary file 3 [file Image_2.JPEG]

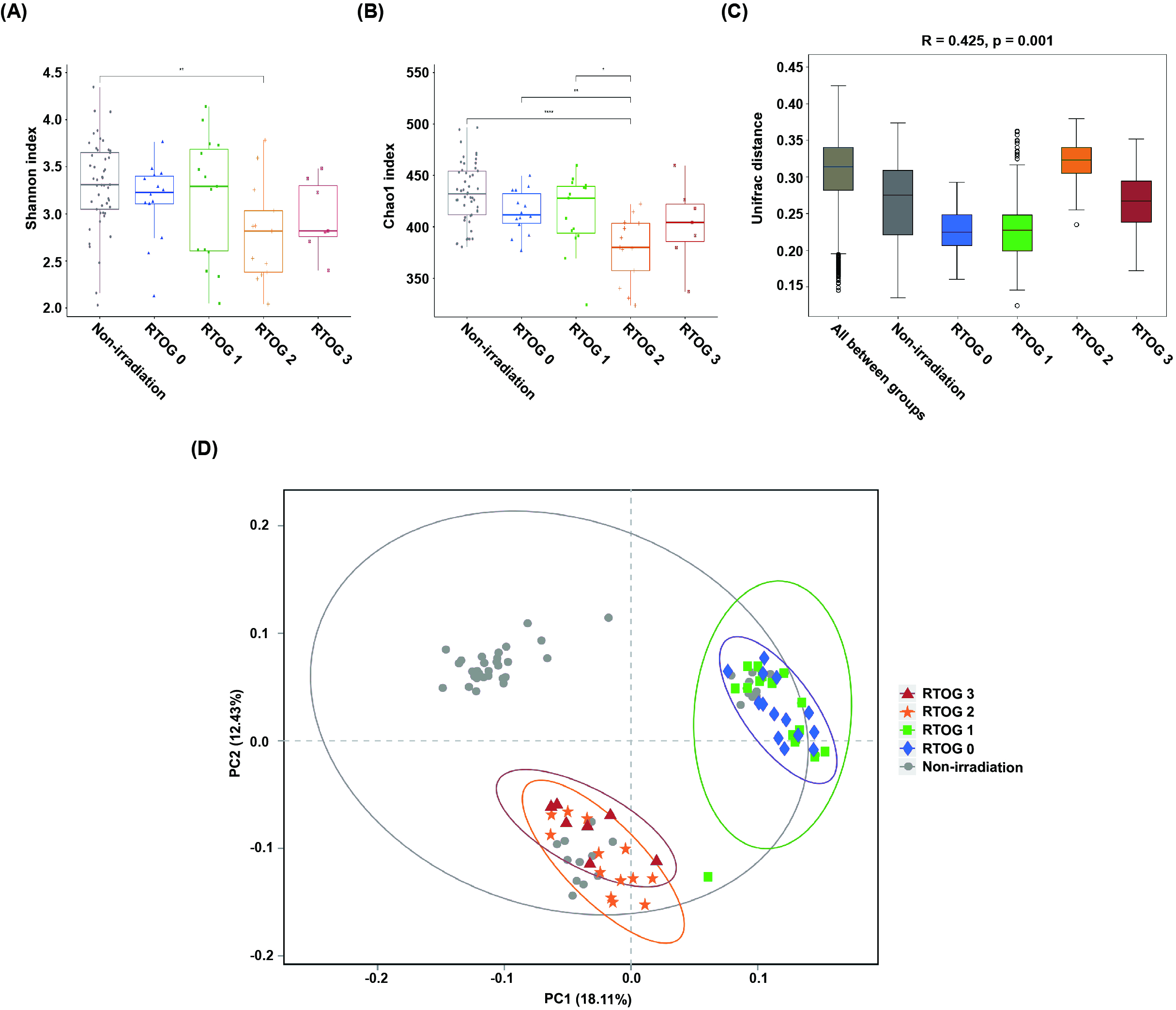

Supplement: Supplementary file 4 [file Image_3.JPEG]

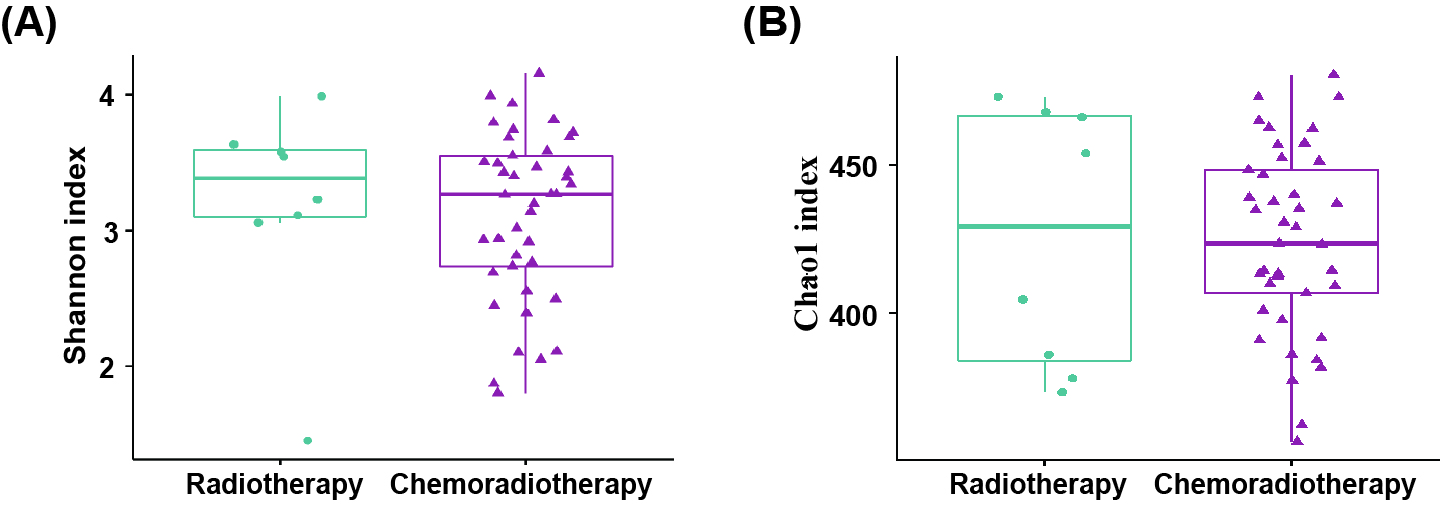

Supplement: Supplementary file 5 [file Image_4.JPEG]

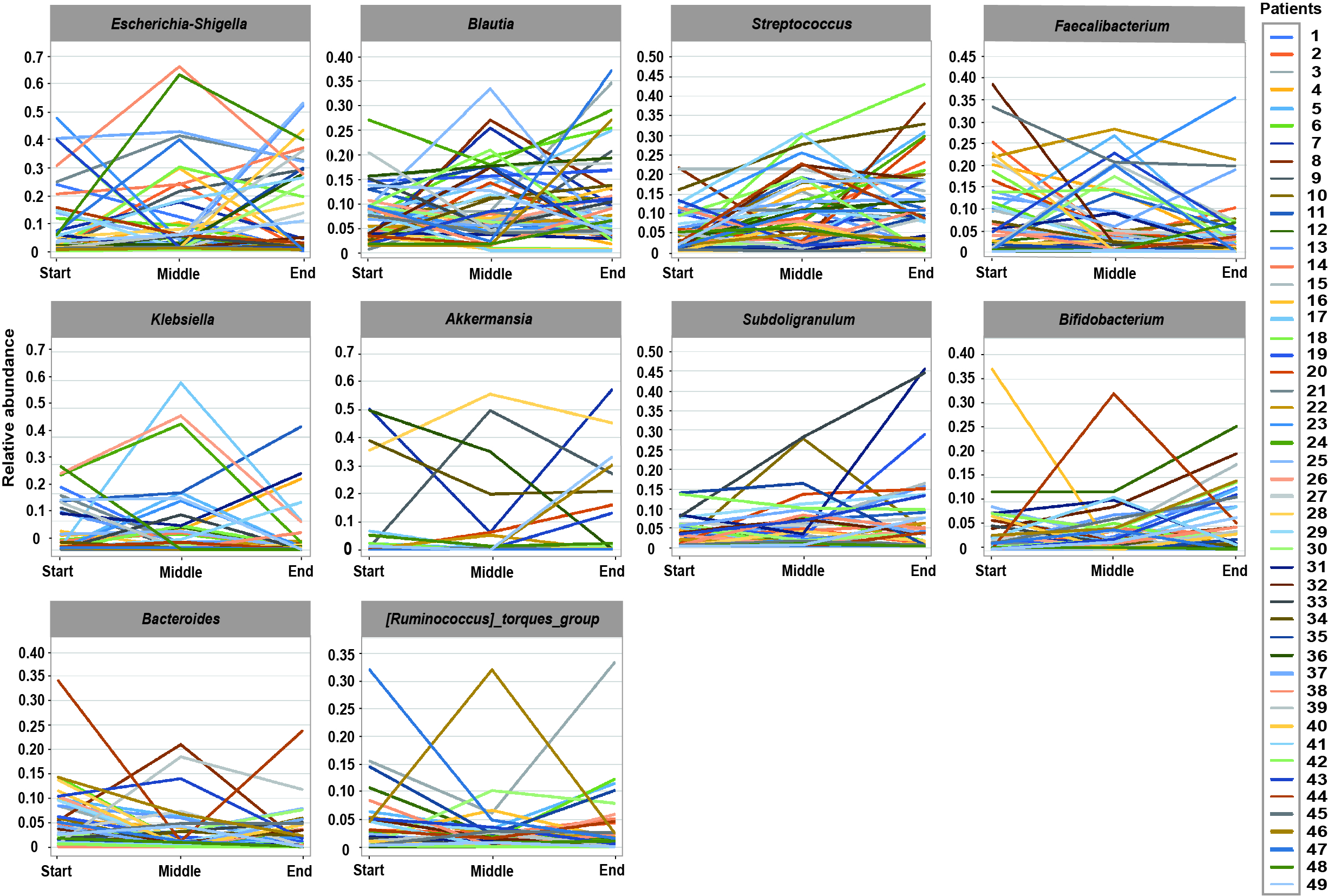

Supplement: Supplementary file 6 [file Image_5.JPEG]

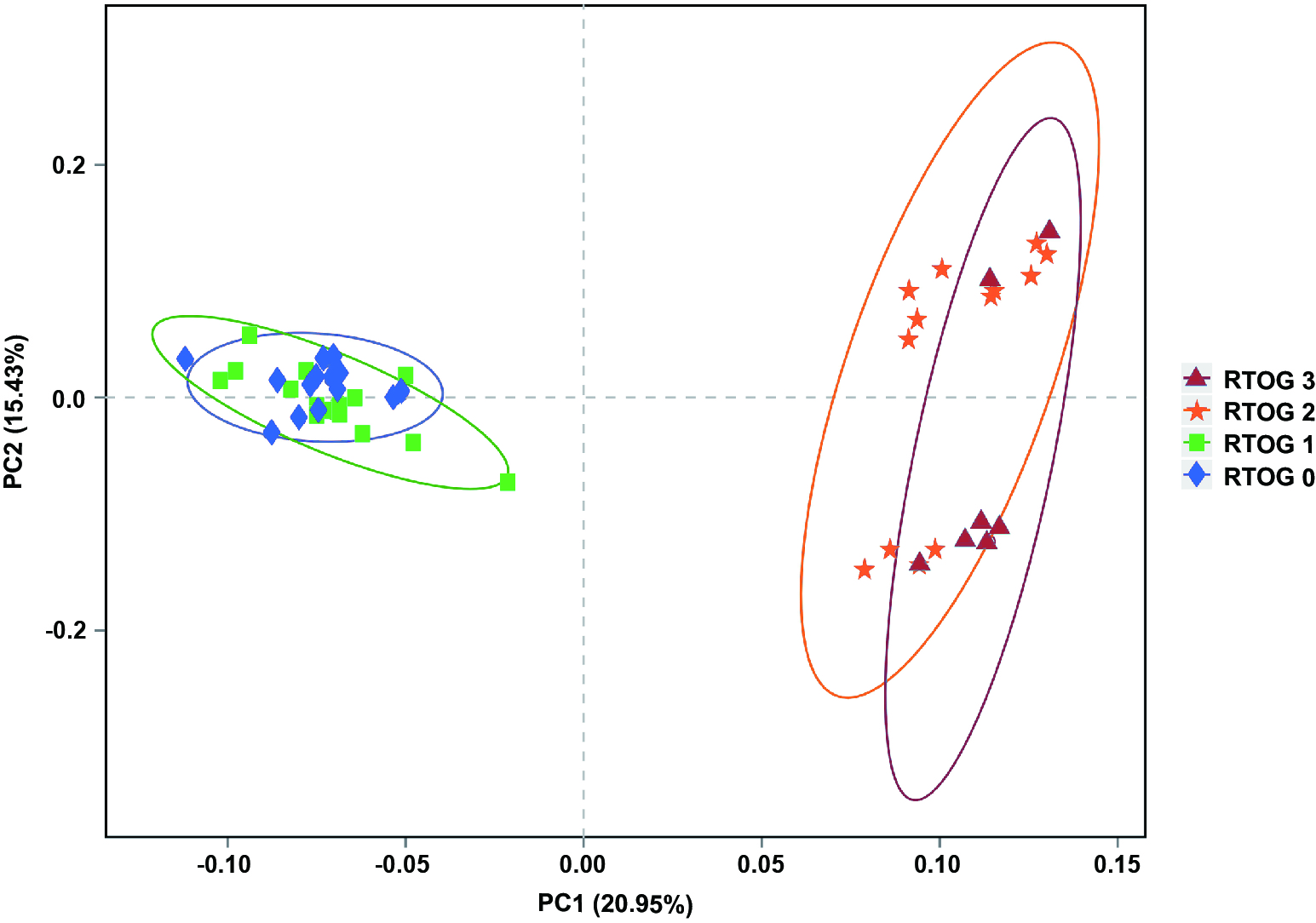

Supplement: Supplementary file 7 [file Image_6.JPEG]

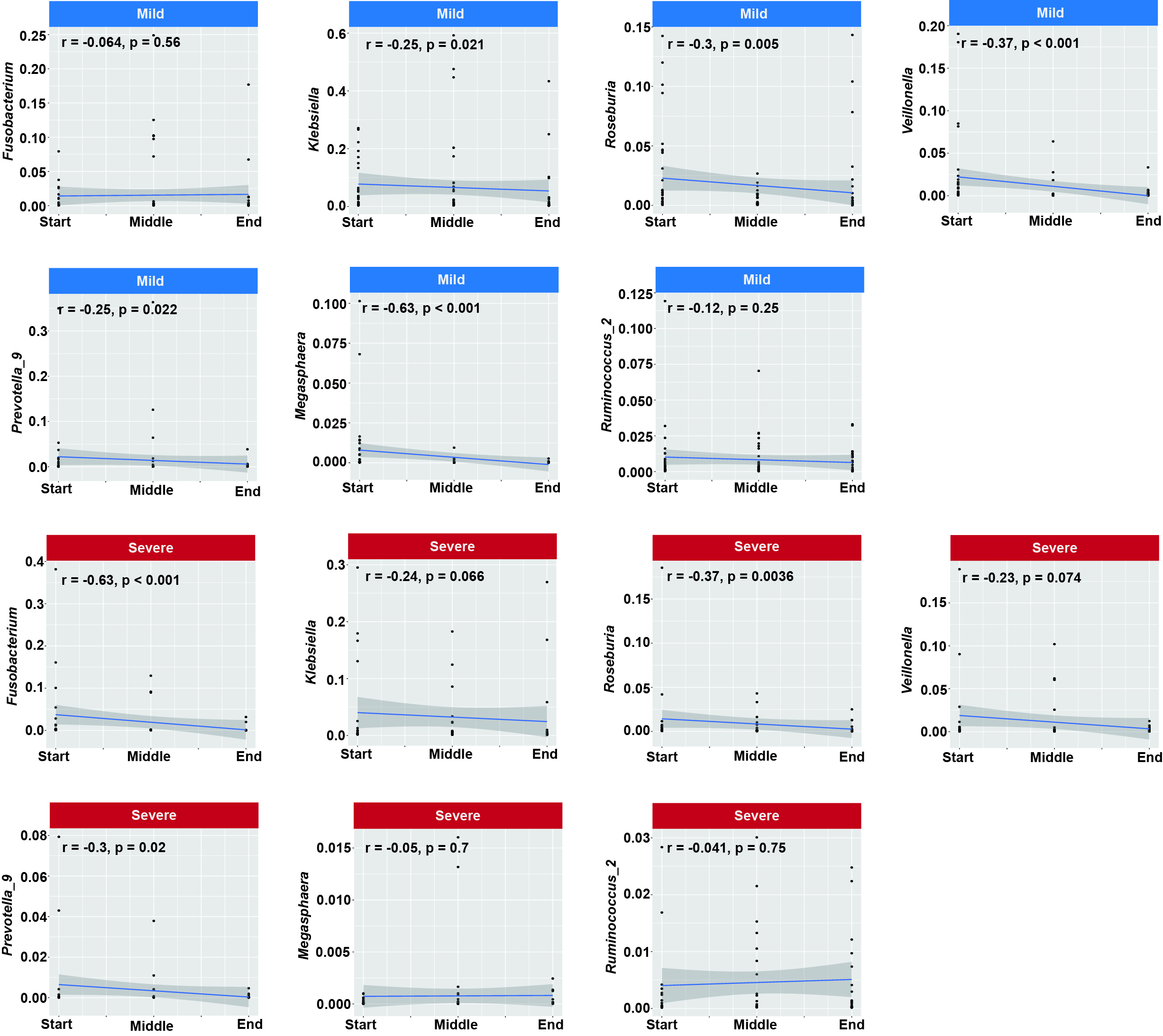

Supplement: Supplementary file 8 [file Image_7.jpg]
